# Supplementary material for: Phylogenetic inference of changes in amino acid propensities with single-position resolution
Source: PLoS Comput Biol. 2022 Feb 18;18(2):e1009878. doi: 10.1371/journal.pcbi.1009878 (PMC9106220; doi:10.1371/journal.pcbi.1009878)
Supplement: S1 File — Fig A in S1 File. Phylogenetic tree of B and C HIV-1 subtypes used in simulation analysis. Green clade is subtype B; blue clade is subtype C; black dots are the two strains to which we applied our test in simulation analysis; red dot is a point of fitness shift. Fig B in S1 File. False Positive Rate of our approach for phylogenetic trees with ~1650, 3300 and 6600 species in simulations for different scaled fitness values of the preferred variant X; black line, median; grey area, 90% confidence interval from random sampling of 1000 amino acids for 100 times. Upper row: all amino acids are considered, bottom row: only testable amino acids are considered. Fig C in S1 File. Performance of the d-test for a phylogenetic tree of subtypes B and C with all branches squeezed or stretched 2 or 4 times, in simulations for different scaled fitness values of the preferred variant. Red line, median True Positive Rate (TPR); blue line, median False Positive Rate (FPR). 90% confidence bands were obtained by random subsampling of 1000 amino acids in 100 trials. Upper row: all amino acids are considered, bottom row–only testable amino acids are considered. Fig D in S1 File. Various conditions for simulations described in Tables C, D in S2 File. Blue dots—focal nodes, red dots—alternative points of shift in fitness vectors. Fig E in S1 File. Schematic representation of cases that can decrease performance of the d-test. Red dots, focal nodes; blue cross, point of fitness shift. A, ideal case; B, both focal points have the same fitness vector, but fitness shift occurs closer to one of them, leading to false positives; C, there is a long branch between the fitness shift and one of the focal points, leading to false negatives; D, distance between the two focal points is less than the mean distance between one of them and species with the same fitness landscape, leading to false negatives. Fig F in S1 File. Performance of the d-test for a phylogenetic tree of Metazoa and Fungi (Fig D (B)) o [file pcbi.1009878.s001.pdf]

Supplementary Figures

Phylogenetic inference of changes in amino acid propensities with single-position resolution

Galya V. Klink[1], Olga V. Kalinina[2], Georgii A. Bazykin[3,1,*]

[1]Institute for Information Transmission Problems (Kharkevich Institute) of the Russian Academy of Sciences, Moscow, Russia

[2]Helmholtz Institute for Pharmaceutical Research Saarland (HIPS), Helmholtz Centre for Infection Research (HZI), Saarbrücken, Germany; Medical Faculty, Saarland University, Homburg, Germany

[3]Skolkovo Institute of Science and Technology, Skolkovo, Russia

*Corresponding author: Georgii A. Bazykin, e-mail: g.bazykin@skoltech.ru

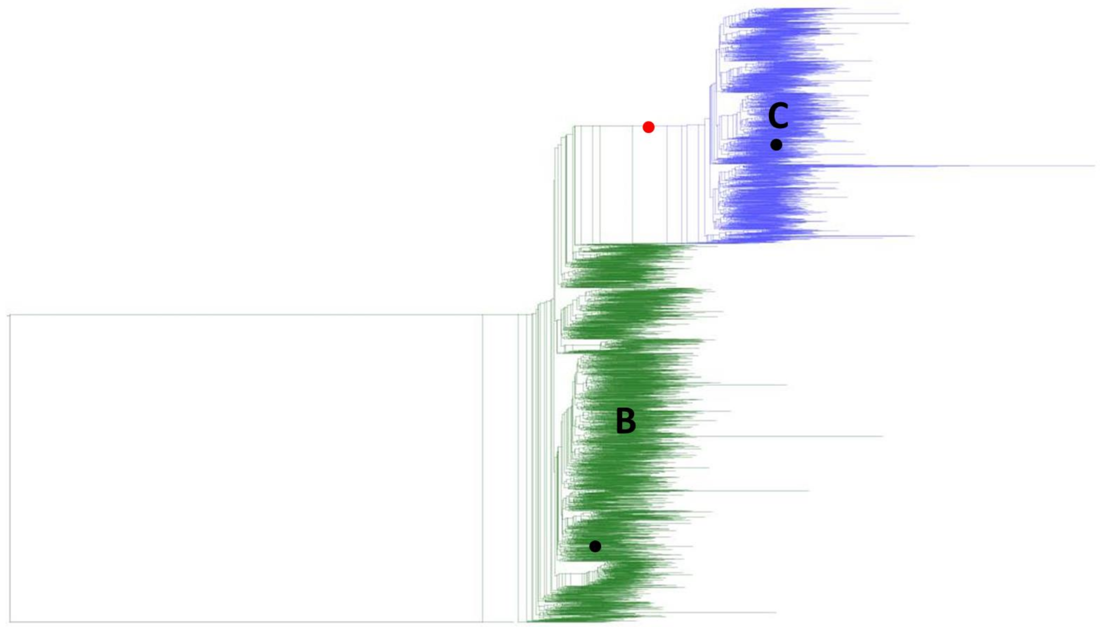

Fig A. Phylogenetic tree of B and C HIV-1 subtypes used in simulation analysis. Green clade is subtype B; blue clade is subtype C; black dots are the two strains to which we applied our test in simulation analysis; red dot is a point of fitness shift.

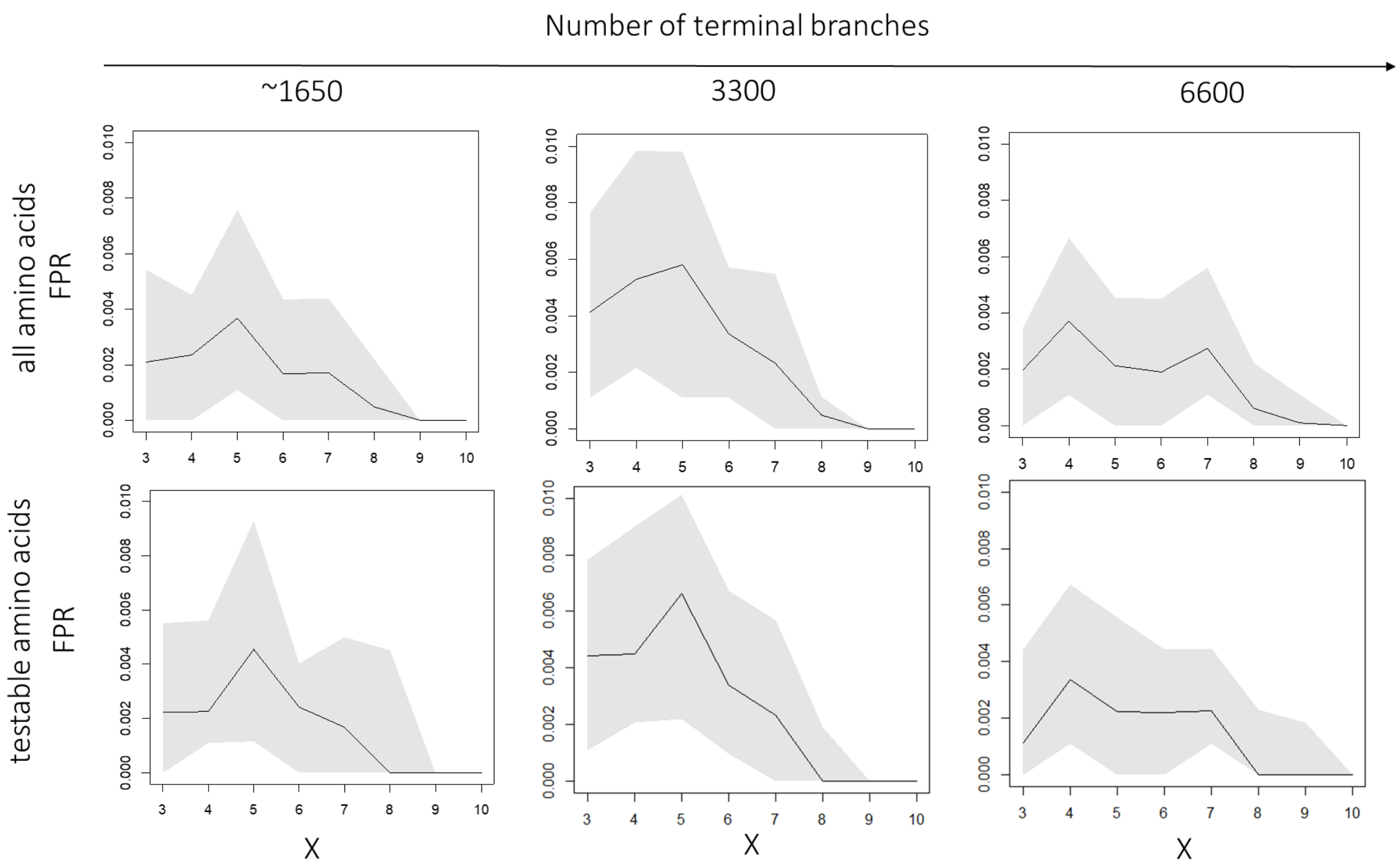

Fig B. False Positive Rate of our approach for phylogenetic trees with ~1650, 3300 and 6600 species in simulations for different scaled fitness values of the preferred variant *X;* black line, median; grey area, 90% confidence interval from random sampling of 1000 amino acids for 100 times. Upper row: all amino acids are considered, bottom row: only testable amino acids are considered.

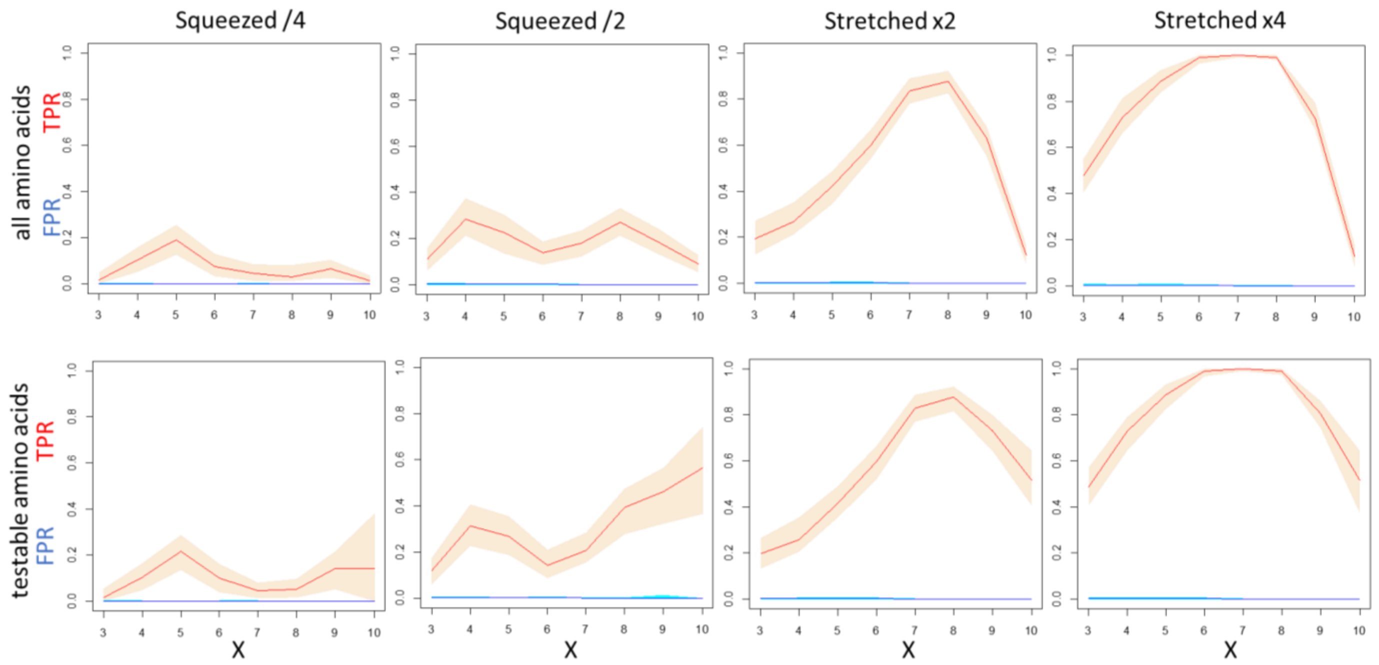

Fig C. Performance of the d-test for a phylogenetic tree of subtypes B and C with all branches squeezed or stretched 2 or 4 times, in simulations for different scaled fitness values of the preferred variant. Red line, median True Positive Rate (TPR); blue line, median False Positive Rate (FPR). 90% confidence bands were obtained by random subsampling of 1000 amino acids in 100 trials. Upper row: all amino acids are considered, bottom row – only testable amino acids are considered.

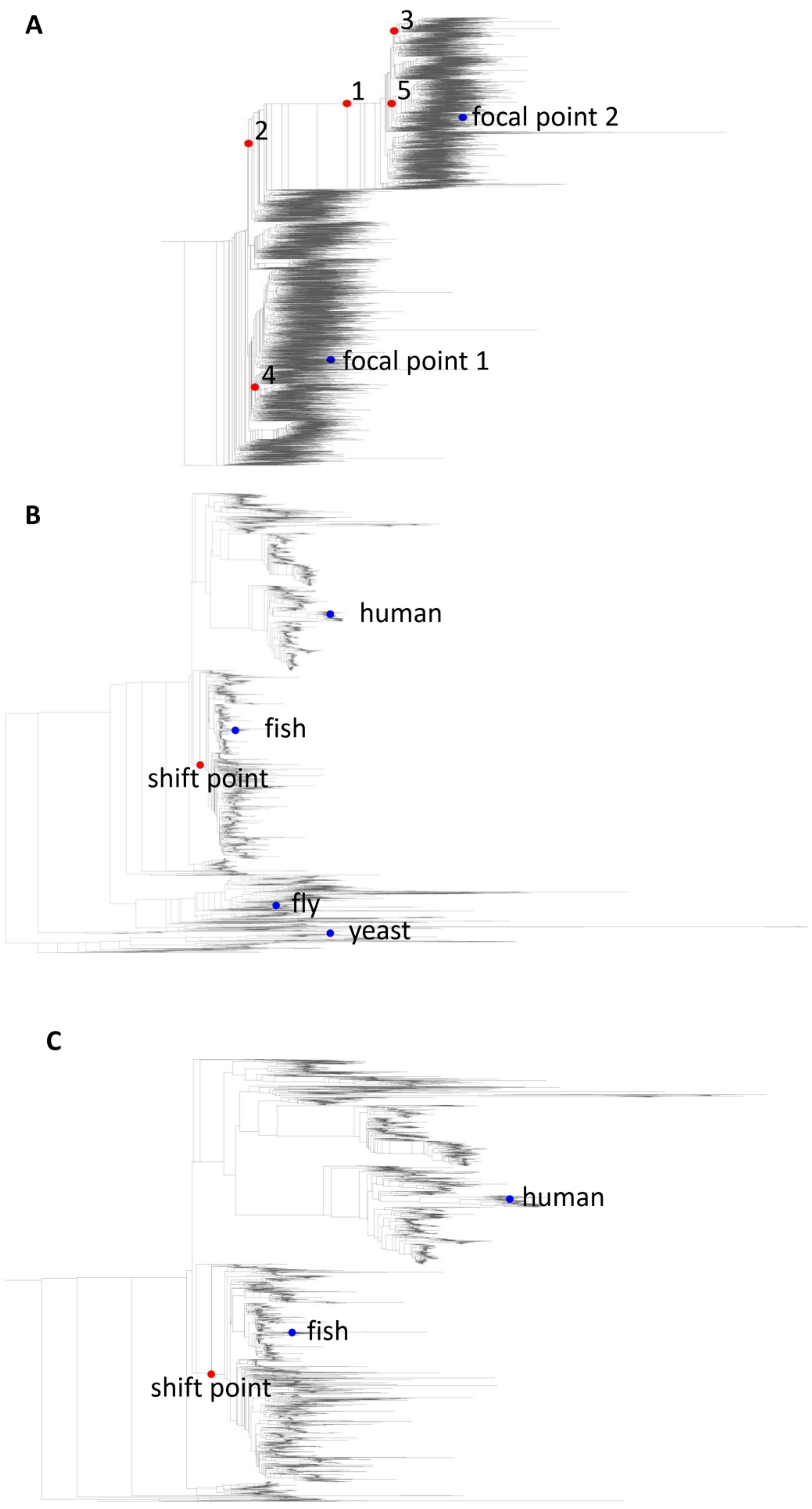

Fig D. Various conditions for simulations described in Tables C, D in S2 File. Blue dots - focal nodes, red dots - alternative points of shift in fitness vectors.

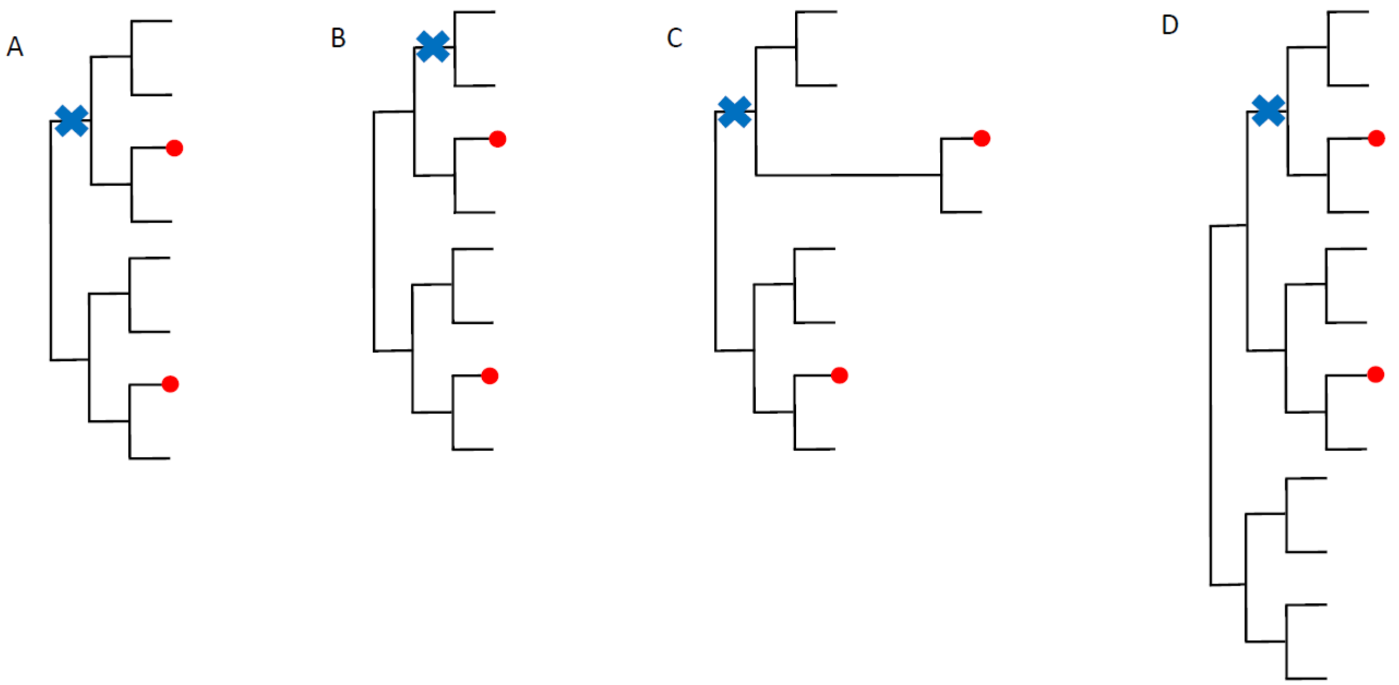

Fig E. Schematic representation of cases that can decrease performance of the d-test. Red dots, focal nodes; blue cross, point of fitness shift. **A**, ideal case; **B**, both focal points have the same fitness vector, but fitness shift occurs closer to one of them, leading to false positives; **C**, there is a long branch between the fitness shift and one of the focal points, leading to false negatives; **D**, distance between the two focal points is less than the mean distance between one of them and species with the same fitness landscape, leading to false negatives.

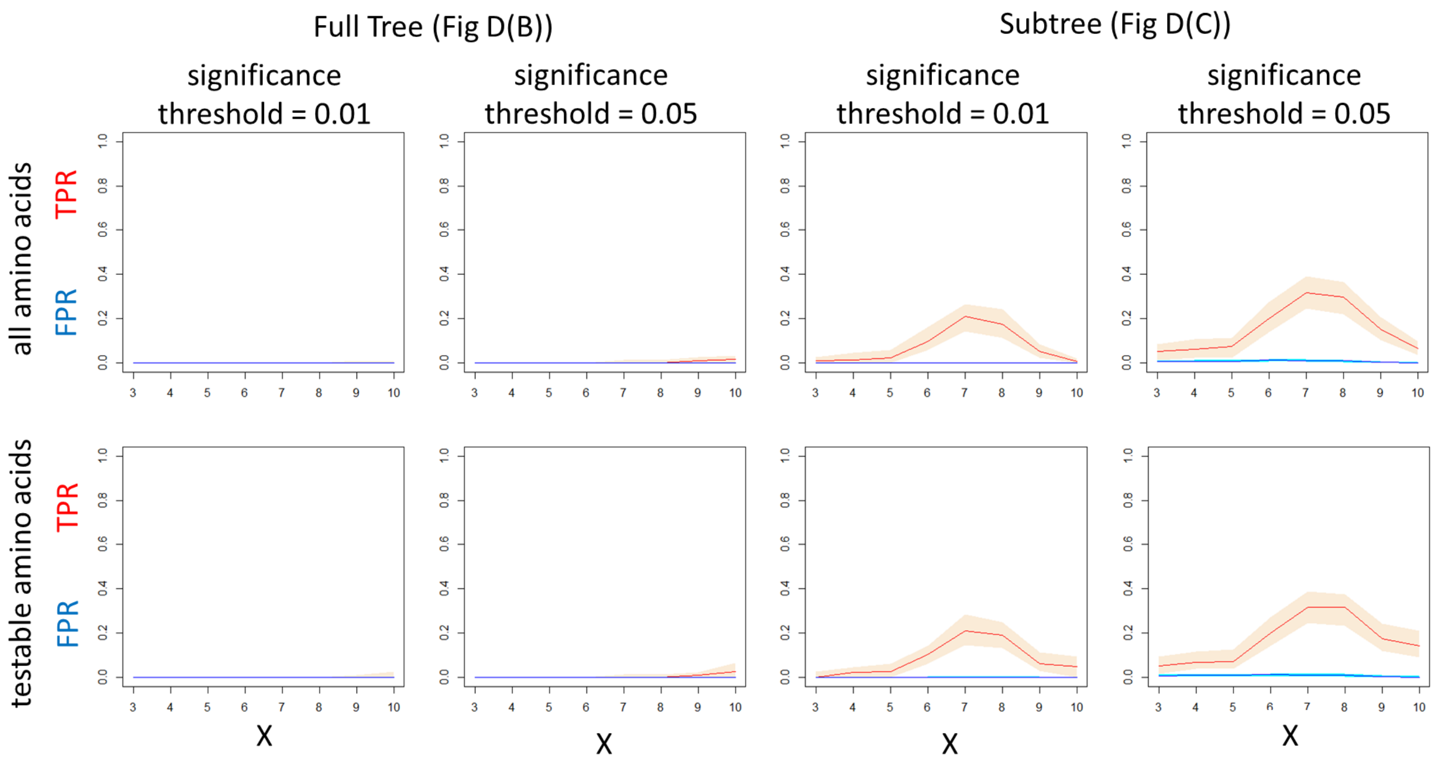

Fig F. Performance of the d-test for a phylogenetic tree of Metazoa and Fungi (Fig D(B)) or its subtree for Vertebrates (Fig D(C)), in simulations for different scaled fitness values of the preferred variant. Red line, median True Positive Rate (TPR); blue line, median False Positive Rate (FPR). 90% confidence bands were obtained by random subsampling of 1000 amino acids in 100 trials. Upper row: all amino acids are considered, bottom row – only testable amino acids are considered.

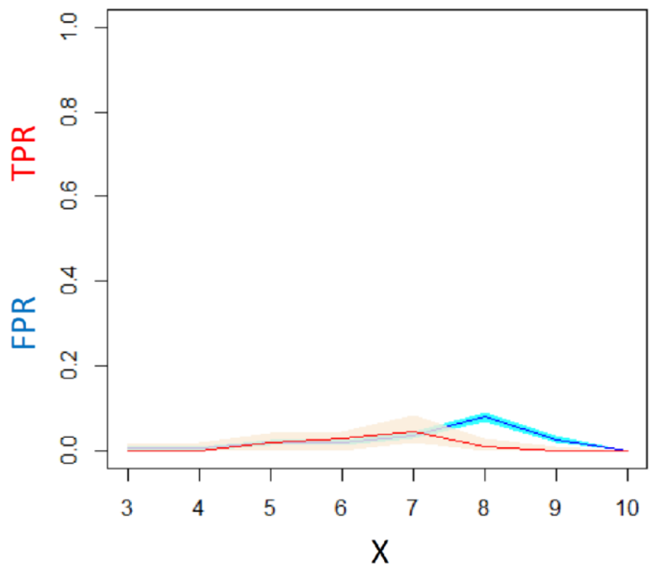

Fig G. Performance of GEMME for a phylogenetic tree of subtypes B and C, in simulations for different scaled fitness values of the preferred variant. An amino acid in a site was considered significant if it has positive score in one subtype and negative score in the other subtype. Red line, median True Positive Rate (TPR); blue line, median False Positive Rate (FPR). 90% confidence bands were obtained by random subsampling of 1000 amino acids in 100 trials.

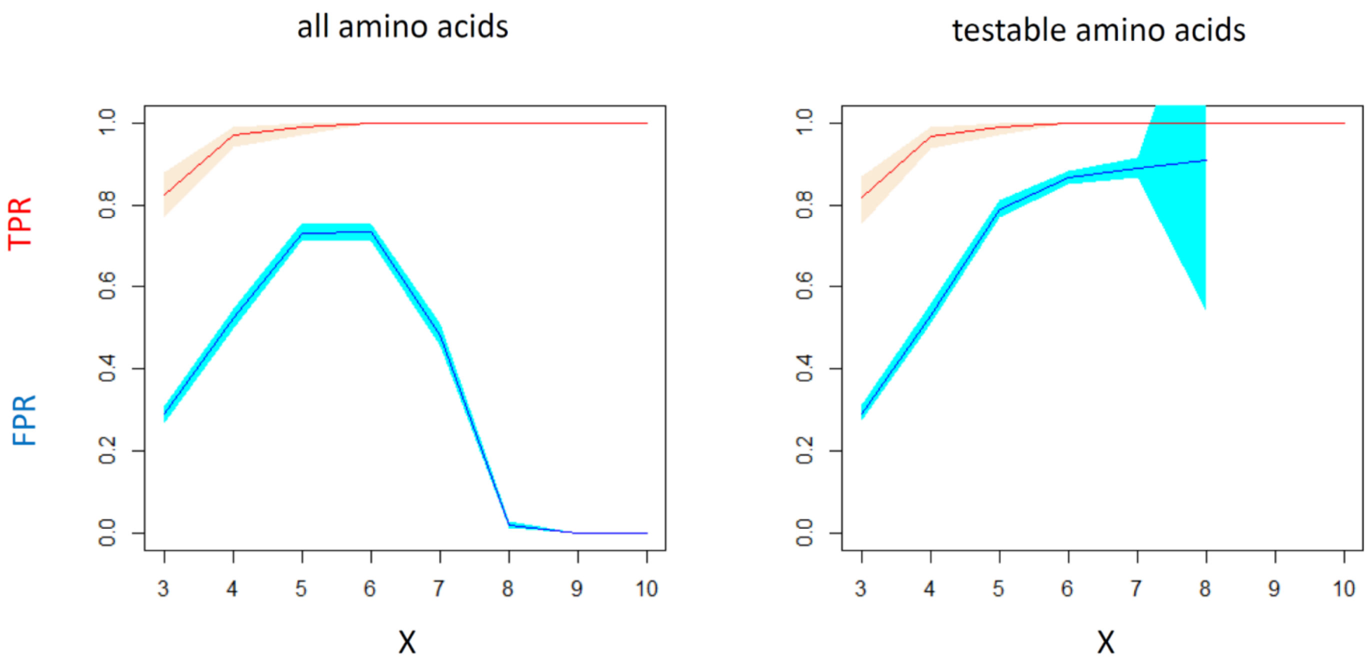

Fig H. Performance of prevalence-based test for a phylogenetic tree of subtypes B and C, in simulations for different scaled fitness values of the preferred variant. Red line, median True Positive Rate (TPR); blue line, median False Positive Rate (FPR). 90% confidence bands were obtained by random subsampling of 1000 amino acids in 100 trials.

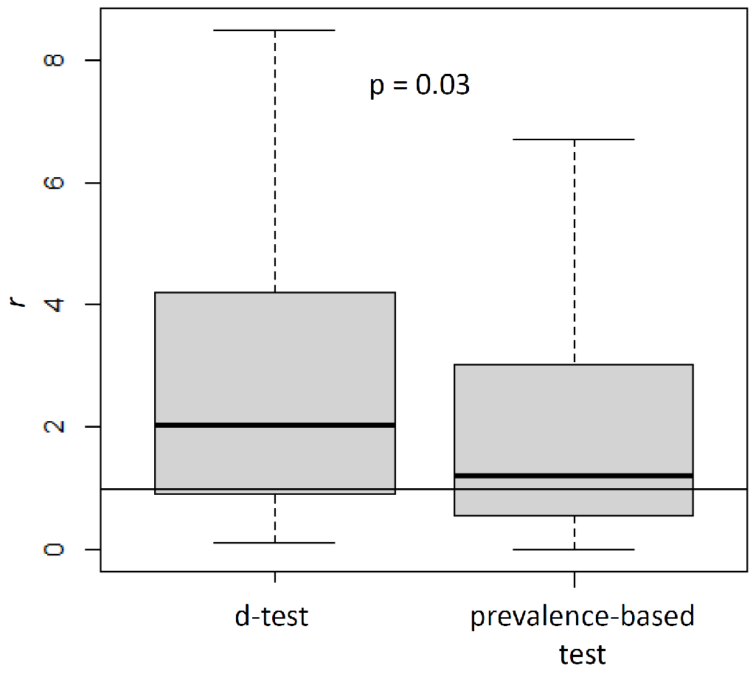

Fig I. Ratio of experimentally measured fitness is subtype for which amino acid is proximal and subtype for which amino acid is distal is higher for amino acids that are significant in d-test than amino acids that are significant in prevalence-based test. Significance value of two-sided Wilcoxon rank sum test is shown.

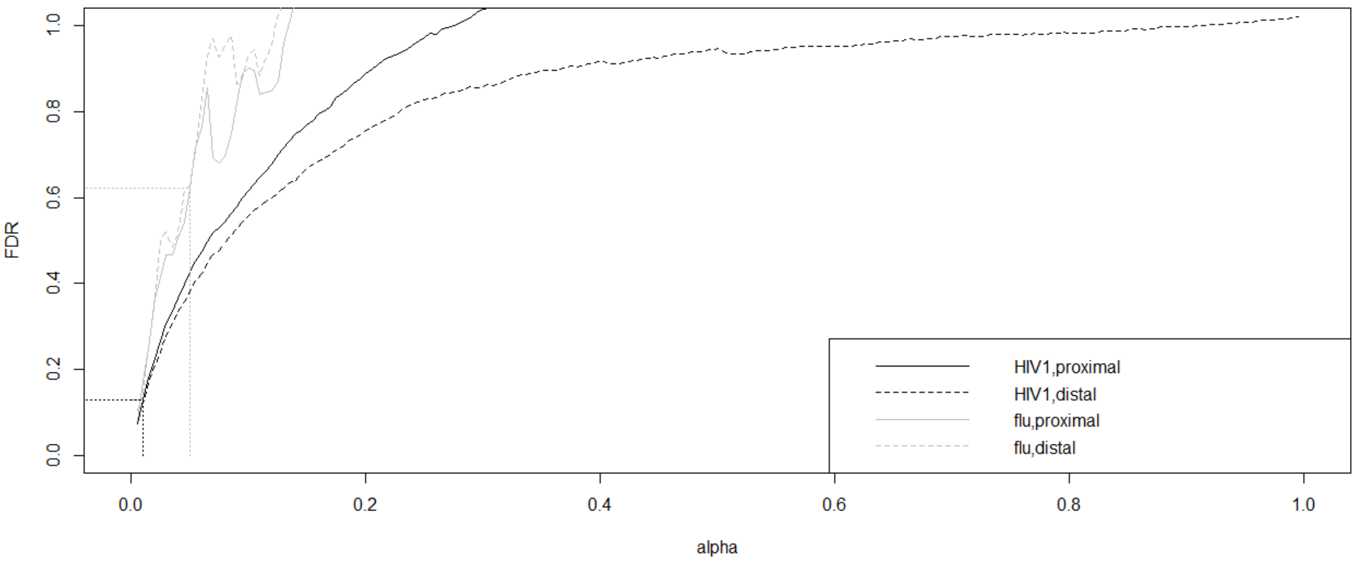

Fig J. Q-value for different significance thresholds obtained as min (α*P)/V, where α is the significance threshold, P is the number of testable amino acids at this α, and V is the number of significant tests at this α, in testing of the hypothesis that an amino acid is proximal or distal for one focal node. Black, gp160; grey, HA; solid, hypothesis of proximity; dashed, hypothesis of distality. Dotted lines correspond to the significance threshold used in the main analysis for gp160 (black) and HA (grey).
