# Supplementary material for: Phylogenetic inference of changes in amino acid propensities with single-position resolution
Source: PLoS Comput Biol. 2022 Feb 18;18(2):e1009878. doi: 10.1371/journal.pcbi.1009878 (PMC9106220; doi:10.1371/journal.pcbi.1009878)
Supplement: S2 File — Table A in S2 File. Power of d-test in simulations with different selective constraints for three sample sizes (median values of TPR and FPR for 100 random subsamples of 1000 amino acids). Table B in S2 File. Power of d-test in simulations with different selective constraints for four evolutionary rates (median values of TPR and FPR for 100 random subsamples of 1000 amino acids). Table C in S2 File. Performance of the d-test under different simulation conditions. Phylogenetic tree with points of fitness shift is shown in Fig D (A) in S1 File. *—cases when fitness shift occurred in a phylogenetic neighborhood of one focal node, but both focal nodes had the same fitness vector. Fitness of amino acids with changing preferences is shown in red. Sensitivity and specificity that maximized Yoden’s coefficient (TPR-FPR) and therefore may serve as the “optimal” performance are shown in “max (Yoden’s coeff)” column. The performance was calculated across all sites of the protein using only testable amino acids. Table D in S2 File. Power of d-test in simulations with different selective constraints for mitochondrial tree (Fig D (B) in S1 File) and its subtree (Fig D (C) in S1 File) (median values of TPR and FPR for 100 random subsamples of 1000 amino acids). Table E in S2 File. Some of variable fitness amino acids are located in sites with different antigenicity in viral subtypes. Table F in S2 File. Consensus amino acids in a pair of viral subtypes in sites with variable fitness amino acids that reside in epitopes of antibodies with different binding capacity in two subtypes. (DOCX) [file pcbi.1009878.s002.docx]

**Supplementary Tables**

**Phylogenetic inference of changes in amino acid propensities with single-position resolution**

Galya V. Klink^1^, Olga V. Kalinina^2^, Georgii A. Bazykin^3,1,*^

^1^Institute for Information Transmission Problems (Kharkevich Institute) of the Russian Academy of Sciences, Moscow, Russia

^2^Helmholtz Institute for Pharmaceutical Research Saarland (HIPS), Helmholtz Centre for Infection Research (HZI), Saarbrücken, Germany; Medical Faculty, Saarland University, Homburg, Germany

^3^Skolkovo Institute of Science and Technology, Skolkovo, Russia

*Corresponding author: Georgii A. Bazykin, e-mail: g.bazykin@skoltech.ru

**Table A.** Power of d-test in simulations with different selective constraints for three sample sizes (median values of TPR and FPR for 100 random subsamples of 1000 amino acids)

|  | TPR | | | FPR | | |
| --- | --- | --- | --- | --- | --- | --- |
| X  tree | 4-9  (mean) | 3 | 10 | 4-9  (mean) | 3 | 10 |
| all amino acids | | | | | | |
| dense | 0.75 | 0.41 | 0.24 | 0.002 | 0.001 | 0 |
| original | 0.43 | 0.29 | 0.1 | 0.003 | 0.004 | 0 |
| sparse | 0.18 | 0.09 | 0.02 | 0.001 | 0.002 | 0 |
| only testable amino acids | | | | | | |
| dense | 0.77 | 0.4 | 0.48 | 0.002 | 0.001 | 0 |
| original | 0.49 | 0.29 | 0.56 | 0.003 | 0.004 | 0 |
| sparse | 0.28 | 0.09 | 0.4 | 0.001 | 0.002 | 0 |

**Table B.** Power of d-test in simulations with different selective constraints for four evolutionary rates (median values of TPR and FPR for 100 random subsamples of 1000 amino acids)

|  | TPR | | | FPR | | |
| --- | --- | --- | --- | --- | --- | --- |
| X  tree | 4-9  (mean) | 3 | 10 | 4-9  (mean) | 3 | 10 |
| all amino acids | | | | | | |
| squeezed x4 | 0.08 | 0.01 | 0.02 | 0.0004 | 0.001 | 0 |
| squeezed x2 | 0.21 | 0.12 | 0.09 | 0.001 | 0.004 | 0 |
| stretched x2 | 0.6 | 0.18 | 0.13 | 0.001 | 0.002 | 0 |
| stretched x4 | 0.86 | 0.49 | 0.14 | 0.002 | 0.004 | 0 |
| only testable amino acids | | | | | | |
| squeezed x4 | 0.11 | 0.02 | 0.17 | 0.0005 | 0.001 | 0 |
| squeezed x2 | 0.29 | 0.12 | 0.44 | 0.002 | 0.004 | 0 |
| stretched x2 | 0.62 | 0.2 | 0.52 | 0.001 | 0.002 | 0 |
| stretched x4 | 0.9 | 0.49 | 0.5 | 0.002 | 0.004 | 0 |

**Table C.** Performance of the d-test under different simulation conditions. Phylogenetic tree with points of fitness shift is shown in Fig D(A) in S1 File. * - cases when fitness shift occurred in a phylogenetic neighborhood of one focal node, but both focal nodes had the same fitness vector. Fitness of amino acids with changing preferences is shown in red. Sensitivity and specificity that maximized Yoden’s coefficient (TPR-FPR) and therefore may serve as the “optimal” performance are shown in “max(Yoden’s coeff)” column. The performance was calculated across all sites of the protein using only testable amino acids.

| **simulations with fitness shift** | | | | | | |
| --- | --- | --- | --- | --- | --- | --- |
| FitnessVector1 | FitnessVector2 | ShiftPoint | α=0.01 | | max(Yoden’s coeff) | |
|  |  |  | sensitivity | specificity | sensitivity | specificity |
| (8,1,1,1,1,1,1,1,1,1,1,1,1,1,1,1,1,1,1,1) | (1,8,1,1,1,1,1,1,1,1,1,1,1,1,1,1,1,1,1,1) | 1 | 0.58 | 0.9993 | 0.85 | 0.95 |
| (8,1,1,1,1,1,1,1,1,1,1,1,1,1,1,1,1,1,1,1) | (1,8,1,1,1,1,1,1,1,1,1,1,1,1,1,1,1,1,1,1) | 2 | 0.07 | 1 | 0.39 | 0.89 |
| (8,1,1,1,1,1,1,1,1,1,1,1,1,1,1,1,1,1,1,1) | (1,8,1,1,1,1,1,1,1,1,1,1,1,1,1,1,1,1,1,1) | 3* | - | 0.91 | - | - |
| (8,1,1,1,1,1,1,1,1,1,1,1,1,1,1,1,1,1,1,1) | (1,8,1,1,1,1,1,1,1,1,1,1,1,1,1,1,1,1,1,1) | 4 | 0.6 | 0.99 | 0.83 | 0.92 |
| (8,1,1,1,1,1,1,1,1,1,1,1,1,1,1,1,1,1,1,1) | (1,8,1,1,1,1,1,1,1,1,1,1,1,1,1,1,1,1,1,1) | 5 | 0.79 | 0.994 | 0.86 | 0.97 |
| (8,8,1,1,1,1,1,1,1,1,1,1,1,1,1,1,1,1,1,1) | (1,1,8,8,1,1,1,1,1,1,1,1,1,1,1,1,1,1,1,1) | 1 | 0.42 | 0.9946 | 0.75 | 0.8 |
| (8,8,1,1,1,1,1,1,1,1,1,1,1,1,1,1,1,1,1,1) | (1,8,1,1,1,1,1,1,1,1,1,1,1,1,1,1,1,1,1,1) | 1 | 1 | 0.864 | 1 | 0.9 |
| (8,1,8,1,1,1,1,1,1,1,1,1,1,1,1,1,1,1,1,1) | (1,8,8,1,1,1,1,1,1,1,1,1,1,1,1,1,1,1,1,1) | 1 | 0.97 | 0.992 | 0.99 | 0.98 |
| (8,1,2,3,4,5,6,7,1,1,1,1,1,1,1,1,1,1,1,1) | (1,8,2,3,4,5,6,7,1,1,1,1,1,1,1,1,1,1,1,1) | 1 | 0.76 | 0.992 | 0.9 | 0.9 |
| (8,1,2,3,4,5,6,7,8,1,1,1,1,1,1,1,1,1,1,1) | (1,8,2,3,4,5,6,7,8,1,1,1,1,1,1,1,1,1,1,1) | 1 | 0.74 | 0.995 | 0.87 | 0.96 |
| (8,1,1.1,1.2,1.3,1.4,1.5,1.6,1.7,1,1,1,1,1,1,1,1,1,1,1) | (1,8,1.1,1.2,1.3,1.4,1.5,1.6,1.7,1,1,1,1,1,1,1,1,1,1,1) | 1 | 0.61 | 0.997 | 0.87 | 0.94 |
| **simulations with constant fitness** | | | | | | |
| (8,1,1,1,1,1,1,1,1,1,1,1,1,1,1,1,1,1,1,1) | - | - | - | 0.9991 | - | - |
| (8,8,1,1,1,1,1,1,1,1,1,1,1,1,1,1,1,1,1,1) | - | - | - | 1 | - | - |
| (8,1,2,3,4,5,6,7,1,1,1,1,1,1,1,1,1,1,1,1) | - | - | - | 0.9993 | - | - |
| (8,8,1,2,3,4,5,6,7,8,1,1,1,1,1,1,1,1,1,1) | - | - | - | 0.9972 | - | - |
| (8,1,1.1,1.2,1.3,1.4,1.5,1.6,1.7,1,1,1,1,1,1,1,1,1,1,1) | - | - | - | 0.997 | - | - |

**Table D.** Power of d-test in simulations with different selective constraints for mitochondrial tree (Fig D(B) in S1 File) and its subtree (Fig D(C) in S1 File) (median values of TPR and FPR for 100 random subsamples of 1000 amino acids)

|  |  | TPR | | | FPR | | |
| --- | --- | --- | --- | --- | --- | --- | --- |
| X  tree | p-value threshold | 4-9  (mean) | 3 | 10 | 4-9  (mean) | 3 | 10 |
| all amino acids | | | | | | | |
| Tree2 | 0.01 | 0 | 0 | 0 | 0 | 0 | 0 |
| Tree3 | 0.01 | 0.1 | 0.01 | 0.007 | 0.0004 | 0 | 0 |
| Tree2 | 0.05 | 0.002 | 0 | 0.02 | 0 | 0 | 0 |
| Tree3 | 0.05 | 0.19 | 0.05 | 0.07 | 0.008 | 0.008 | 0.001 |
| only testable amino acids | | | | | | | |
| Tree2 | 0.01 | 0 | 0 | 0 | 0 | 0 | 0 |
| Tree3 | 0.01 | 0.1 | 0 | 0.05 | 0.0006 | 0.002 | 0 |
| Tree2 | 0.05 | 0.002 | 0 | 0.03 | 0 | 0 | 0 |
| Tree3 | 0.05 | 0.19 | 0.05 | 0.14 | 0.009 | 0.008 | 0 |

**Table E.** Some of variable fitness amino acids are located in sites with different antigenicity in viral subtypes.

| antibody | epitope | PDB id | number of positions in the epitope* | of them, sites with variable fitness amino acids for subtypes A and B | of them, sites with variable fitness amino acids for subtypes B and C | Wilcoxon two-sided test for IC50 in subtypes A and B | Wilcoxon two-sided test for IC50 in subtypes B and C |  |
| --- | --- | --- | --- | --- | --- | --- | --- | --- |
| Doria-Rose et al., 2012 | | | | | | | |  |
| VRC01 | CD4-binding site | 3NGB | 42 | 2 | 13 | 0.09 | 0.23 |  |
| PG9 | V1/V2 loop | 3U4E | 13 | 1 | 2 | **0.030**** | **0.004** |  |
| Georgiev et al., 2013 | | | | | | | |  |
| VRC01 | CD4-binding site | 3NGB | 42 | 2 | 13 | **0.005** | **0.010** |  |
| NIH45-46 |  | 4JKP | n/a | n/a | n/a | 0.83 | 0.05439 |  |
| b12 |  | 2NY7 | 54 | 6 | 15 | **0.0002** | **0.002** |  |
| PG9 | V1/V2 loop | 3U4E | 13 | 1 | 2 | **0.0002** | **0.0003** |  |
| 2G12 | glycans in the outer domain of gp120 | 6E5P | 5 | 0 | 2 | **0.023** | **0.00000004** |  |
| 2F5 | gp41 | n/a | n/a | n/a | n/a | 0.936 | **0.00008** |  |
| 4E10 |  | n/a | n/a | n/a | n/a | 0.233 | 0.779 |  |
| PGT121 | outer domain of gp120 and the V3 loop base | 5CEZ | 20 | 0 | 2 | 0.826 | 0.935 |  |
| PGT128 |  | 5C7K | 21 | 0 | 2 | 0.399 | 0.076 |  |

*According to [37]

**p-values less than 0.05 are shown in **bold**

**Table F**. Consensus amino acids in a pair of viral subtypes in sites with variable fitness amino acids that reside in epitopes of antibodies with different binding capacity in two subtypes.

| **A-B comparison** | | | | |
| --- | --- | --- | --- | --- |
| antibody | higher affinity to subtype | site (hxb2 numbering) | amino acid in A strain | amino acid in B strain |
| VRC01 | A | 281 | A | A |
| VRC01 | A | 425 | N | N |
| b12 | B | 178 | R | K |
| b12 | B | 181 | I | V |
| b12 | B | 281 | A | A |
| b12 | B | 425 | N | N |
| b12 | B | 426 | M | M |
| b12 | B | 430 | V | V |
| PG9 | A | 168 | K | K |
| **B-C comparison** | | | | |

| antibody | higher affinity to subtype | site (hxb2 numbering) | amino acid in B strain | amino acid in C strain |
| --- | --- | --- | --- | --- |
| b12 | B | 178 | K | R |
| b12 | B | 179 | L | L |
| b12 | B | 181 | V | I |
| b12 | B | 182 | V | V |
| b12 | B | 183 | P | P |
| b12 | B | 192 | R | R |
| b12 | B | 198 | T | T |
| b12 | B | 281 | A | A |
| b12 | B | 384 | Y | Y |
| b12 | B | 386 | N | N |
| b12 | B | 421 | K | K |
| b12 | B | 426 | M | M |
| b12 | B | 429 | E | E |
| b12 | B | 455 | T | T |
| b12 | B | 471 | G | G |
| PG9 | C | 160 | N | N |
| PG9 | C | 168 | K | K |
| 2G12 | B | 332 | N | N |
| 2G12 | B | 339 | N | N |
