# Supplementary material for: Phylogenetic inference of changes in amino acid propensities with single-position resolution
Source: PLoS Comput Biol. 2022 Feb 18;18(2):e1009878. doi: 10.1371/journal.pcbi.1009878 (PMC9106220; doi:10.1371/journal.pcbi.1009878)
Supplement: S3 File — (PDF) [file pcbi.1009878.s003.pdf]

Robustness of the results of the d-test for different focal nodes and for random subsets

We performed this analysis for the highest-likelihood of the ten trees that we created for our main analysis described in the manuscript.

First, we performed the d-test for ten randomly chosen pairs of focal strains where one strain belonged to subtype A and the other belonged to subtype B, and compared the lists of variable fitness amino acids at 0.01 significance level. 77 of 114 (67.5%) of amino acids that were significant at least in one trial were significant in all ten trials (Fig A). The robustness was higher for those amino acids for which the changes in propensity were the strongest. Indeed, among those amino acids with the difference between distal and proximal standard scores averaged over the significant trials higher than eight, 42 of 43 (97.7%) were significant in all ten trials (Fig B).

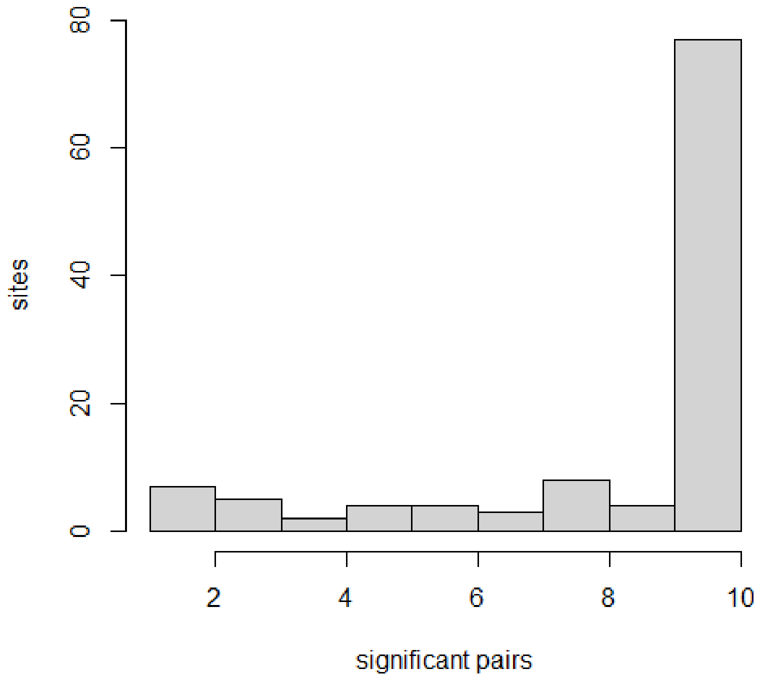

Fig A. Numbers of amino acids that were significant in different numbers of trials, where each trial represented a different pair of focal strains.

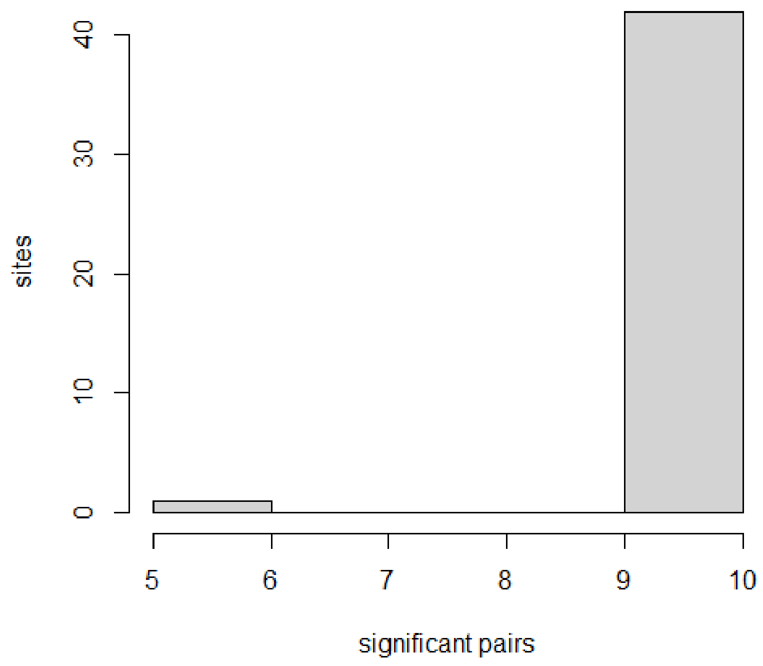

Fig B. Numbers of amino acids that were significant in different numbers of trials, where each trial represented a different pair of focal strains. Only amino acids with the difference between distal and proximal standard scores averaged over the significant trials higher than 8 are shown.

Second, we performed a d-test for ten subsets of sequences obtained by taking 50% of samples randomly. 94 of 112 (83.9%) of amino acids that were significant at least in one trial were significant in all ten trials (Fig C). 49 of 50 (98%) of amino acids with the difference between distal and proximal standard scores averaged over the significant trials higher than eight were significant in all ten trials (Fig D).

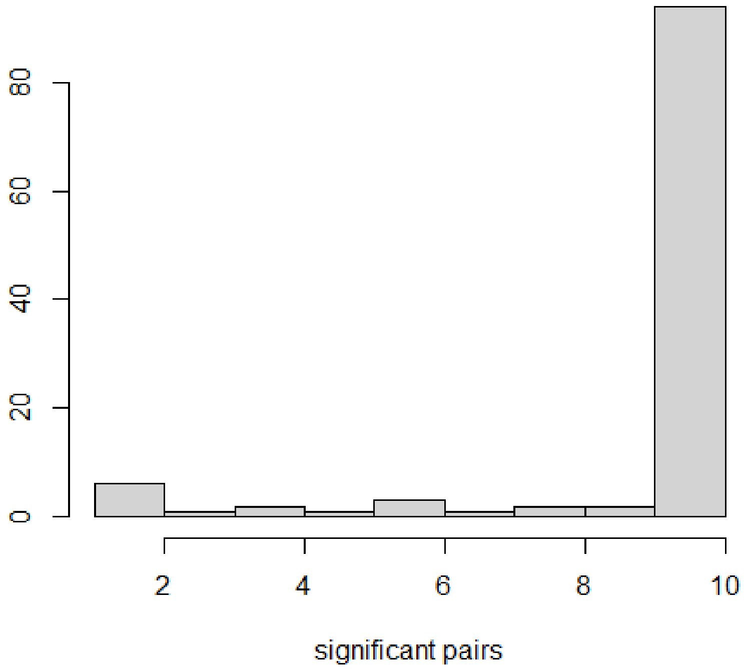

Fig C. Number of amino acids that were significant in different number of trials, where each trial represented a randomly chosen 50% of data.

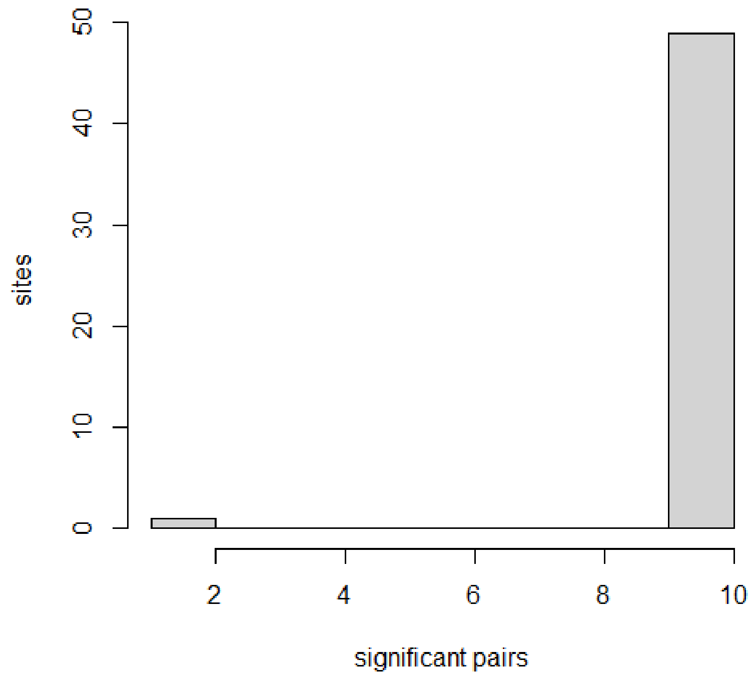

Fig D. Number of amino acids that were significant in different number of trials, where each trial represented a randomly chosen 50% of data. Only amino acids with the difference between distal and proximal standard scores averaged over the significant trials higher than 8 are shown.

This analysis shows that the d-test is robust to subsampling, and that its results are similar for different focal species with strongly intersected phylogenetic neighborhoods.
