# Supplementary material for: Phylogenetic inference of changes in amino acid propensities with single-position resolution
Source: PLoS Comput Biol. 2022 Feb 18;18(2):e1009878. doi: 10.1371/journal.pcbi.1009878 (PMC9106220; doi:10.1371/journal.pcbi.1009878)
Supplement: Attachment — Submitted filename: Review_Phylogenetic_Inference_AA_Propensities.pdf [file pcbi.1009878.s005.pdf]

Review – Phylogenetic inference of changes in amino acid propensities with single-position resolution

The authors address the problem of finding instances where the fitnesses of amino acids have changed during the evolutionary history of a gene family. Their method, the d-test, is notable for being able to detect these changes at a single site without having to fit highly parametric methods on very large alignments. The method works by first reconstructing ancestral sequences at each node, then determining whether the position of substitutions into each amino acid is biased to be closer to one of two pre-determined lineages. The authors validate this test by simulating amino acid sequences on an HIV phylogeny, finding low false positive rates and fitness-dependent false negative rates. They also ground-truth their method with two empirical cases (HIV *env* and influenza HA) which are subject to frequent episodes of elevated dN/dS. They compare their amino acid fitness changes to dN/dS results from codon models, to published values from Deep Mutational Scanning (DMS), and to immunogenicity data.

This is a simple, elegant, and likely relatively fast test that could be used on a large scale. The method is especially well validated due to the inclusion of the DMS data, which supports its results experimentally.

The main suggestion I have is that the suite of simulations discussed in Supplementary Figure S3 and Supplementary Table S4 should be brought into the main text and discussed more fully, including providing full Methods for them. These are very important for understanding the potential use cases of the d-test in different phylogenies with different evolutionary histories, and for correctly interpreting the sensitivity and specificity measures reported in the main text (i.e., these are for a specific type of phylogeny and shift position). Note that they provide some important reassurances, such as the fact that the method is not confounded when multiple amino acids change fitness at once.

Otherwise, some specific notes by line number:

---

32. Abstract. 'Fitness conferred by the same allele may differ between genotypes' – should also mention environment, e.g. different host species as you do in the intro

141. Perhaps add a sentence describing what models the SELVa software implements.

166. Could the sensitivity be improved if substitutions towards the base of the tree were given more weight? It seems that the method does not account for A->A 'substitutions' later on in the tree, that is if there is a substitution early on in the tree that is not undone, then there must be many mutations introduced that would have fixed if neutral but that instead go extinct against the fitter A background.

167. For the terms 'moderate', 'stronger', 'weaker' selection – would be useful to know how often we should expect real selection coefficients to have these values? Possibly relate to studies like Tamuri et al. 2014 Genetics, Rodrigue et al. 2010 PNAS.

179. Does the number of non-testable AAs depend on the mutation rate?

200. The simulations in Supplementary Figure 3 and Supp Table 2 need to be discussed in the main text. It is not clear from the captions of Table 2 exactly what was done here.

- Were there 100 replicates, as in the main simulations?

- Were false positives calculated the same way when there were multiple AAs changing fitness?
- Why were trees 2 and 3 chosen as test cases?
- How were the fitness vectors chosen for each case, and what was the rationale?

209. Throughout the ms, please clarify when you are referring to amino acid types (e.g A, R, Y) versus amino acid sites, i.e. positions in an AA sequence.

253. What was the control?

261-2. “sites having gaps closer than five positions more than in 5% of the data set” – meaning unclear, do you mean all AA sites within 5 bases of a gap?

350. I don’t have Table S3 in my copy of the manuscript.

480. “it is not sensitive to differences in the overall substitution rate…” not sure what the evidence for this is, did you simulate mutation rate variation among lineages?

514. I imagine it holds as long as large shifts in propensities are relatively rare?

603-4. Perhaps also cite dos Reis, Tamuri et al. 2011 Mol Biol Evol.

626. Perhaps include a sentence mentioning that the method requires an ancestral sequence reconstruction as input.

722. Please include methods for Supp Table 2 and Supp Fig 3.

727-732. Please clarify ‘proportion of amino acids’ – does this mean the proportion out of all amino acid types, pooled across all 500 sites

Supp Table 1. Please change left column to ‘Dense, Original, Sparse’ to match text.

Supp Table 2. See comment to line 200 above.
