# Supplementary material for: Phylogenetic inference of changes in amino acid propensities with single-position resolution
Source: PLoS Comput Biol. 2022 Feb 18;18(2):e1009878. doi: 10.1371/journal.pcbi.1009878 (PMC9106220; doi:10.1371/journal.pcbi.1009878)
Supplement: Attachment — Submitted filename: Response_to_reviewers.docx [file pcbi.1009878.s006.docx]

**Reviewer #1:** The article presents a method to estimate fitness changes for amino-acids in fixed positions between subtypes/clades. It is presented as a general method to be applied to protein families but tested exclusively on viral proteins.

The method is of interest but I found that the presentation is not straightforward, the testing is limited, the method is not described clearly, the comparison with tools that estimate phenotypic effects is not performed.

Some remarks:

- Protein families are not presented precisely: sequence length, number of sequences in the set, sequence conservation level, conservation levels and number of sequences within the clades containing focal points used in the article….

**We now provide extended information about the proteins that we used, giving all the details requested. Considering the number of sequences within the clades containing focal points used, it is important to note that our method does not rely on specific predefined clades; instead, it requires no arbitrary decisions about what clade size to consider, and considers the phylogenetic neighborhoods of focal species. In case of HIV-1 with distinct subtypes separated by long branches, phylogenetic neighborhoods of focal species from subtypes A and B coincide with the corresponding subtypes.**

- As mentioned above, the approach is presented as a general approach, applicable to arbitrary protein families but tested on viral proteins only. This should be discussed and if the authors think that the method can be applied to other protein families, they should say it and provide testing. The problem might be to find available experimental data allowing for a comparative evaluation of the fitness within different clades. If this is the case, then a comparison with computationally generated data is important. See below.

**As our simulations show, our method is applicable to any proteins with sufficient variability, although its performance is sensitive to the structure of the phylogenetic tree. The constraints of the method are discussed in detail in the Discussion.**

- Overall the writing is unclear with notions that are not completely standard. For instance, what is a “segment” of a phylogenetic tree (pp4)? Is it a branch point? Maybe use more common notions.

**We revised the paper, hopefully clarifying the writing. In the fragment referred to by the Reviewer, we now changed “segment” to “part”. Here, we try to give the intuition behind the approach; the formal definitions for our metrics are provided in the following sentences. We have revised this section for clarity.**

Other examples are:

pp 28. The phrase “by adding, to each terminal node of the initial tree, two new branches”… what does this mean? How general this way to do is? Can you envisage some other operation?

**The goal of this procedure was to double the number of tips of the tree, so as to test how the sensitivity and specificity of our method depends on the number of considered samples. We now clarified the explanation of this procedure. Below, we test how the performance of our method depends on other aspects of our dataset.**

pp29. The vector defined as {X,1,….,1,1,1} is not a way to write it! The 20 “1”s correspond to what? I counted the number of “1”s btw and some wording could be added. How does this vector evolve? A formal description of your method is needed. It would make the whole message much clearer.

**We apologize for the typo - it should have been 19 “1”s rather than 20, indicating the scaled fitness of each of the 19 amino acids other than the only one which has scaled fitness *X*. We now consistently use the ordered set notation, and clarify in the text what the vector corresponds to. How the vector evolves is described in detail in the “Method validation with simulations and data” Section.**

- There is no comparison with the matrix reconstruction of DMS experiments proposed by other groups. The method identifies a subset of significant positions in a sequence and correlates them with DMS measures. Today, we have methods reconstructing matrices from DMS experiments that perform very well. See, for instance, besides Hopf et al. 2017 and Riesselman et al. 2018 also

Laine, E., Karami, Y., & Carbone, A. (2019). GEMME: a simple and fast global epistatic model predicting mutational effects. Molecular biology and evolution, 36(11), 2604-2619.

Munro, D., & Singh, M. (2020). DeMaSk: a deep mutational scanning substitution matrix and its use for variant impact prediction. Bioinformatics, 36(22-23), 5322-5329.

The model introduced by the authors should be compared to the performance of these methods. They are based on different ideas and it is important that they discuss them. Especially the GEMME method, based on trees, could be considered and discussed on the methodological site. The authors seem to ignore these developments. The model introduced in this work could gain in impact if the author can show its interest in others predictions.

**It is a difficult task to directly compare our method to these other methods. First, these methods predict the effect of a mutation by assessing the fitness of an amino acid as compared to the wild type in a particular species. By contrast, our method is aimed at detecting changes in fitness of an amino acid between phylogenetic neighborhoods of two focal species. Second, they do not calculate statistical significance. To make the methods comparable as requested by the Reviewer, we now introduce the standard score for our d-statistic, calculated as its distance from the expectation calculated in number of standard deviations (standard score). For the HIV-1 dataset, we show that the difference between the standard scores of the d-statistic in subtypes A and B is significantly correlated with the difference between the GEMME scores for the two focal species (Spearman correlation test, rho = 0.24, p < 2.2e-16) as well as with the difference between DeMask scores for the two focal species (Spearman correlation test, rho = 0.11, p = 7.048e-16). The fact that the correlation is not perfect is perhaps unsurprising, as these methods are expected to have the highest power for different types of amino acid sites. GEMME and DeMask account for the physico-chemical similarity of amino acids and directly depend on site conservation. By contrast, our method does not directly depend on these site properties. As a result, it may still find differences in fitness effects of amino acids at sites that are less conservative in terms of GEMME. These new results are now provided and discussed.**

- As discussed, in this article there is no reference to the genetic context provided by the entire sequence that in principle could be relevant for predicting the phenotype as demonstrated in the works above. This point should be discussed on the light of precise computational comparisons.

**Indeed, similarly to methods such as GEMME, we do not explicitly model epistasis between sites. Such epistasis however would contribute to our signal, as more phylogenetically related samples are also more similar in sequence. We now discuss this.**

- A careful combined analysis of parameters like the size of the clades and their variability in sequence identity/similarity should be realized.

**In the revision, we substantially expand the Validation section of the manuscript by adding new analyses.**

- The quality of the figures is very poor. Small points with indistinguishable colors.

**We have revised the figures, hopefully making them better.**

**Reviewer #2:**

The authors address the problem of finding instances where the fitnesses of amino acids have changed during the evolutionary history of a gene family. Their method, the d-test, is notable for being able to detect these changes at a single site without having to fit highly parametric methods on very large alignments. The method works by first reconstructing ancestral sequences at each node, then determining whether the position of substitutions into each amino acid is biased to be closer to one of two pre-determined lineages. The authors validate this test by simulating amino acid sequences on an HIV phylogeny, finding low false positive rates and fitness-dependent false negative rates. They also ground-truth their method with two empirical cases (HIV env and influenza HA) which are subject to frequent episodes of elevated dN/dS. They compare their amino acid fitness changes to dN/dS results from codon models, to published values from Deep Mutational Scanning (DMS), and to immunogenicity data.

This is a simple, elegant, and likely relatively fast test that could be used on a large scale. The method is especially well validated due to the inclusion of the DMS data, which supports its results experimentally.

**We thank the Reviewer for the high assessment of our work and of its validation on experimental data.**

The main suggestion I have is that the suite of simulations discussed in Supplementary Figure S3 and Supplementary Table S4 should be brought into the main text and discussed more fully, including providing full Methods for them. These are very important for understanding the potential use cases of the d-test in different phylogenies with different evolutionary histories, and for correctly interpreting the sensitivity and specificity measures reported in the main text (i.e., these are for a specific type of phylogeny and shift position). Note that they provide some important reassurances, such as the fact that the method is not confounded when multiple amino acids change fitness at once.

**We now substantially extended the corresponding section in the main text.**

Otherwise, some specific notes by line number:

32. Abstract. ‘Fitness conferred by the same allele may differ between genotypes’ – should also

mention environment, e.g. different host species as you do in the intro

**This is correct, thank you. We now add “and environments” in the Abstract**

141. Perhaps add a sentence describing what models the SELVa software implements.

**This is described in the “Method validation with simulations and data” section of the Results, and in the “Simulated evolution” section of Methods. We now made these descriptions more detailed.**

166. Could the sensitivity be improved if substitutions towards the base of the tree were given more weight? It seems that the method does not account for A->A ‘substitutions’ later on in the tree, that is if there is a substitution early on in the tree that is not undone, then there must be many mutations introduced that would have fixed if neutral but that instead go extinct against the fitter A background.

**Taking into account the size of clades that are occupied by the same amino acids is a very good idea. However, it requires a more complicated model as a neutral expectation of the size of such a clade is needed. This is an important venue for future work.**

167. For the terms ‘moderate’, ‘stronger’, ‘weaker’ selection – would be useful to know how often we should expect real selection coefficients to have these values? Possibly relate to studies like Tamuri et al. 2014 Genetics, Rodrigue et al. 2010 PNAS.

**The estimated distributions of selection coefficients vary substantially for different methods and datasets. We now mention several studies in the Discussion, including those proposed by the Reviewer.**

179. Does the number of non-testable AAs depend on the mutation rate?

**Yes, it depends on all factors that influence the number of substitutions and amino acid diversity; now mentioned.**

200. The simulations in Supplementary Figure 3 and Suppl Table 2 need to be discussed in the main text. It is not clear from the captions of Table 2 exactly what was done here.

• Were there 100 replicates, as in the main simulations?

**No, for this table, overall TPR and FPR were calculated. We added this information to its caption and to Methods.**

• Were false positives calculated the same way when there were multiple AAs

changing fitness?

**Yes, each amino acid was considered as a single observation independently of the type of the fitness vector. This is mentioned in Methods now.**

• Why were trees 2 and 3 chosen as test cases?

**We used the mitochondrial tree as it is strongly unbalanced, in contrast to Tree1 which is rather balanced. Tree3 is a part of Tree2 which we used to show that the performance of our test depends on the structure of the phylogenetic tree and can be improved when clades that are distant from both focal species (i.e. cannot be attributed to phylogenetic neighborhood of either of them) are removed.**

• How were the fitness vectors chosen for each case, and what was the rationale?

**We chose X=8 for more complex scenarios as it showed the best performance for a simple fitness vector where one amino acid had fitness X and other 19 amino acids had fitness 1. The space of possible evolutionary scenarios is infinite. In picking the scenarios for analysis, we aimed to test the robustness of our method to specifics of the change of the fitness vector. Specifically, we asked whether a fitness change would still be inferred if two amino acids, rather than just one, have a high fitness; if the fitness of the suboptimal amino acids differs between such amino acids; etc.**

209. Throughout the ms, please clarify when you are referring to amino acid types (e.g A, R, Y)

versus amino acid sites, i.e. positions in an AA sequence.

**Thank you, we fixed this wherever the meaning was ambiguous.**

253. What was the control?

**All amino acids with both p-values more than 0.1 were used as a control. It is mentioned in the previous paragraph and clarified in the capture of Fig. 4.**

261-2. “sites having gaps closer than five positions more than in 5% of the data set” – meaning

unclear, do you mean all AA sites within 5 bases of a gap?

**Yes, we meant that we took only sites with no gaps in the 5 positions before and the 5 positions after. It is clarified now.**

350. I don’t have Table S3 in my copy of the manuscript.

**It is a large table contained in a separate file Supplementary Table 3.xlsx (now Supplementary Table 5.xlsx). We now mentioned it in Supplementary Materials.**

480. “it is not sensitive to differences in the overall substitution rate...” not sure what the evidence for this is, did you simulate mutation rate variation among lineages?

**We mean here that as we look for changes in the proportion of substitutions to a particular amino acid from the same ancestral amino acids, the change in overall mutation/substitution rates (i.e. changes in evolutionary rate of a site) do not influence the results. However, changes in mutation rates between particular amino acids may lead to a spurious signal for changes in the fitness landscape. It is discussed in the manuscript.**

514. I imagine it holds as long as large shifts in propensities are relatively rare?

**It seems to be right in general, whereas we can imagine a situation where the same amino acid may become fit in distant clades in parallel due to convergent changes in environments independently of genetic context.**

603-4. Perhaps also cite dos Reis, Tamuri et al. 2011 Mol Biol Evol.

**We now added this reference.**

626. Perhaps include a sentence mentioning that the method requires an ancestral sequence

reconstruction as input.

**We now mentioned it.**

722. Please include methods for Supp Table 2 and Supp Fig 3.

**We now added information about these simulations in Methods.**

727-732. Please clarify ‘proportion of amino acids’ – does this mean the proportion out of all amino acid types, pooled across all 500 sites

**That is right. We clarified it now.**

Supp Table 1. Please change left column to ‘Dense, Original, Sparse’ to match text.

**We fixed it now.**

Supp Table 2. See comment to line 200 above.

**Done.**

**Reviewer #3:** In this manuscript, the authors proposed a statistical d-test to identify changing propensity of amino acid states across phylogeny, by measuring the distance of certain types of substitution to focal taxa in the tree. Proximation to one taxon and distality to another indicate increase of propensity, and hence fitness, of the resulting amino acid to the former clade. The authors use simulation to show its sensitivity and specificity, then show that the detected substitutions are consistent with fitness change measured by deep mutational scanning, while not all of them are under positive selection. The authors then describe the relation between d-test detected variable fitness amino acids with immunogenicity in HIV and Influenza viruses.

Since the test depend heavily on the amino acid states appearing at each site, although the authors have made significant efforts in analyzing the statistical properties of the test, I am still concerned with how stable the test result is, i.e. for the same set of data, how variable the detection power and the accuracy is:

(1) To conduct d-test, two or more focal nodes needs to be selected for measuring d. With different focal nodes assigned for the same two clades, would d-test detect the same set of variable fitness amino acids?

**The more phylogenetic neighborhoods of two alternative focal species intersect, the more similar results these species will give when opposed to the third focal species.**

**To address this comment, we now chose 10 pairs of random species from subtypes A and B in our dataset and compared the results of the d-test between them. The results of ten trials were in agreement. This analysis is included in the manuscript now (see Supplementary Note 1).**

(2) Ten independently reconstructed trees were used in one d-test, but with the same set of taxa. If the propensity change is true for a clade, then variation of taxa included in a clade (not necessarily changing the number of taxa) should not affect the detection results. Is this true?

**We took a subset of 50% random species from our dataset 10 times and conducted the d-test with each subset. Sets of variable fitness amino acids were strongly overlapping. This analysis is included in the manuscript now (see Supplementary Note 1).**

To confirms the superiority of the d-test, there should be a baseline method for control. For example, if one simply compares the observed frequency of amino acid states in each clade and test the significance by some randomized null distribution, how would the performance be worse than the d-test?

**We thank the Reviewer for this suggestion. We now added the analysis of a simple prevalence-based test for changes in amino acid fitness. Its statistics for an amino acid at a site is (A-B)/(A+B), where A and B are numbers of strains containing this amino acid in subtypes A and B, respectively. The null distribution is obtained by 1000 reshuffling trials of amino acid identities in the alignment column. If the left p-value is low, the amino acid is considered as close to subtype A; if the right p-value is low, to subtype B. We show that the d-test outperforms this prevalence-based test both for simulation and data.**

Around Line 371-404, it is argued that the variation of fitness is associated with difference in antigenic binding. However, there is no statistical significance.

**This chapter serves as an illustration of potential biological inference of changes in amino acid propensities. We haven’t seen a significant enrichment of variable fitness amino acids in epitopes that show differentially binding vs constant binding with BnAbs in a pair of subtypes. Nevertheless, we have found some examples of potential connection between changes in fitness of amino acids in epitopes and changes in binding strength.**

Specifically, I cannot understand why the binding could be affected if the most prevalent amino acid does not change.

**As any epitope occupies a region in a protein with length of several (up to several dozen) amino acid positions, the affinity may change due to substitutions at some sites even if amino acid identities of other sites remain the same. Nevertheless, this may cause a shift in amino acid propensities at these unchanged sites, although not strong enough to change the identity of the consensus amino acid. This is clarified now.**

Table S4: It seems there are two rows with VRC01 and PG9. I could not find the explanation for their difference. Besides, is there multiple test correction for p-values in this table?

**These are results of two different studies, but subheadings were missing in this table (now Table S6). We fixed it now. No correction for multiple testing was performed for this table.**
